# An Integrated Photonic Biosensing Platform for Pathogen Detection in Aquaculture

**DOI:** 10.3390/s24165241

**Published:** 2024-08-13

**Authors:** Wout Knoben, Siegfried Graf, Florian Borutta, Zerihun Tegegne, Michael Ningler, Arthur Blom, Henk Dam, Kevin Evers, Rens Schonenberg, Anke Schütz-Trilling, Janneke Veerbeek, Roman Arnet, Mark Fretz, Vincent Revol, Thomas Valentin, Christopher R. Bridges, Stephan K. Schulz, Joost van Kerkhof, Anne Leenstra, Farid Orujov, Henk van Middendorp

**Affiliations:** 1 Surfix Diagnostics, Agro Business Park 2, 6708 PW Wageningen, The Netherlandsrens.schonenberg@surfixdx.com (R.S.);; 2 CSEM, Untere Gründlistrasse 1, 6055 Alpnach, Switzerland; 3 TunaTech GmbH, Merowingerplatz 1A, 40225 Düsseldorf, Germany; borutta@tunatech.de (F.B.);; 4 PHIX Photonics Assembly, Hengelosestraat 525, 7521 AG Enschede, The Netherlands; z.tegegne@phix.com (Z.T.);; 5 LRE Medical GmbH, Georg-Brauchle-Ring 89, 80992 München, Germany; ningler@lre.de

**Keywords:** optical biosensors, photonic structures for sensing, interferometry, hybrid integration, point-of-need sensors, aquaculture, pathogen detection, environmental monitoring, food safety and quality, diagnostics

## Abstract

Aquaculture is expected to play a vital role in solving the challenge of sustainably providing the growing world population with healthy and nutritious food. Pathogen outbreaks are a major risk for the sector, so early detection and a timely response are crucial. This can be enabled by monitoring the pathogen levels in aquaculture facilities. This paper describes a photonic biosensing platform based on silicon nitride waveguide technology with integrated active components, which could be used for such applications. Compared to the state of the art, the current system presents improvements in terms of miniaturization of the Photonic Integrated Circuit (PIC) and the development of wafer-level processes for hybrid integration of active components and for material-selective chemical and biological surface modification. Furthermore, scalable processes for integrating the PIC in a microfluidic cartridge were developed, as well as a prototype desktop readout instrument. Three bacterial aquaculture pathogens (*Aeromonas salmonicida*, *Vagococcus salmoninarum*, and *Yersinia ruckeri*) were selected for assay development. DNA biomarkers were identified, corresponding primer-probe sets designed, and qPCR assays developed. The biomarker for *Aeromonas* was also detected using the hybrid PIC platform. This is the first successful demonstration of biosensing on the hybrid PIC platform.

## 1. Introduction

Biosensors are valuable tools in a wide range of application areas, such as medical diagnostics, agri-food, and environmental monitoring [1]. They can contribute to the global ambitions of health and well-being, the abolishment of hunger, and clean water, which are among the 17 Sustainable Development Goals defined by the United Nations (UN) [2].

One of the major challenges of our time and in the years to come is how to provide the growing world population with healthy and nutritious food in a sustainable way. As a source of proteins, aquaculture, which is the process of rearing, breeding, and harvesting of aquatic species in controlled environments, is an important and strongly developing industry [3]. Compared to agriculture and cattle breeding, aquaculture provides food with a high nutritional value with a relatively low carbon footprint, because fish convert feed into protein more efficiently than other animals. Moreover, specific ingredients in fish products such as omega-3 fatty acids contribute to a healthy diet.

According to the UN’s Food and Agriculture Organization, aquaculture is growing faster than any other major food production sector, with 50% of all consumed aquatic food obtained by aquaculture [3]. However, the ever-present threat of a disease outbreak poses a significant risk to aquaculture businesses and the sector as a whole. The World Bank estimated that disease results in a negative economic impact of $6 billion annually for the global aquaculture industry [4]. Reducing the risk and cost associated with disease outbreaks is a major challenge.

In particular, the Atlantic salmon (*Salmo salar*) aquaculture sector faces significant challenges regarding the disease control of pathogens [5,6]. In salmon production facilities, stressful conditions such as high stocking densities, heavy use of formulated feeds, antibiotics, and other pharmaceuticals can have tremendous environmental and economic impact. Within hatchery systems, non-infectious diseases may weaken the immune system of individual fish, and infectious diseases can rapidly spread by pathogens released from an infected fish by mucus, feces, or urine. Most of these pathogens are waterborne infections and are either contagious viral diseases like infectious pancreatic necrosis, infectious salmon anemia, and cardiomyopathy syndrome, or bacterial infections like furunculosis (by *Aeromonas salmonicida*), bacterial kidney disease (by *Renibacterium salmoninarum*), and vibriosis (by *Vibrio anguillarum* and *Vibrio salmonicida*).

Detection at an early stage is critical for timely treatment or removal of diseased fish to contain or isolate outbreaks. Moreover, many of these pathogens are notifiable diseases for the veterinary authorities. Due to the current regime change from flow-through systems to recirculating aquaculture systems, the need for an effective pathogen monitoring system has become even more urgent.

An important part of the solution may come from biosensor-based monitoring of pathogen levels in aquaculture facilities [7]. Detection of pathogen-specific biomarkers can be performed by different biosensing technologies, such as electrochemical or optical biosensors [8,9]. Biosensors based on Photonic Integrated Circuits (PICs) offer advantageous features such as a high analytical sensitivity, the capability of multiplexing and miniaturization, and the suitability for integration in optofluidic devices [10]. Additionally, these sensors offer advantages such as the prospect of label-free detection, real-time measurement, and immunity to electromagnetic interference. Moreover, PICs are manufactured by standard complementary metal-oxide semiconductor (CMOS)-compatible fabrication techniques, which are ideally suited to cost-effective high-volume manufacturing.

To maximize miniaturization and performance, various techniques have been developed for the hybrid integration of active optical components (typically based on III-V compound semiconductors such as GaAs or InP) into silicon-based PIC platforms [11]. One method involves using micro-optical benches, which have already proven to be commercially successful [12]. However, this approach requires assembling multiple components, increasing the optical device’s footprint. Another method mounts the III-V and silicon photonic chips on separate carriers and assembles them through direct butt coupling at the chip edges [13]. While this scheme allows for high performance, integration is limited to the chip edges, the footprint is increased due to separate carriers, and the method is unsuitable for volume production (wafer-scale integration). The final option is hybrid integration via flip-chip bonding, where III-V components are placed on top of or into an etched recess on the silicon-based platform [14]. This technique offers a low footprint, short electrical paths, and effective thermal management by creating direct thermal contact with the silicon substrate. Despite the added assembly complexity, flip-chip assembly can be very cost-effective at higher production volumes.

Previously, a highly promising PIC biosensor platform based on the asymmetric Mach–Zehnder Interferometer (aMZI) was developed and used for detection of biomarkers in different application areas [15,16,17,18,19,20,21]. Biosensor platforms based on aMZI possess advantages such as enhanced sensitivity, compact size, and improved performance in label-free biosensing applications. Moreover, it was demonstrated that a high level of integration could be achieved, with light source and detector components directly flip-chip bonded on a hybrid PIC, which was in turn assembled into a microfluidic cartridge [18]. This hybrid system demonstrated proof of concept, but the fabrication process was cumbersome and not scalable. Moreover, experiments with the hybrid system were limited to measurement of bulk refractive index changes. No biosensing experiments were performed with this system due to mutual compatibility issues between the required chemical and biological surface modification steps and the presence of integrated components on the PIC.

**Figure 1:**
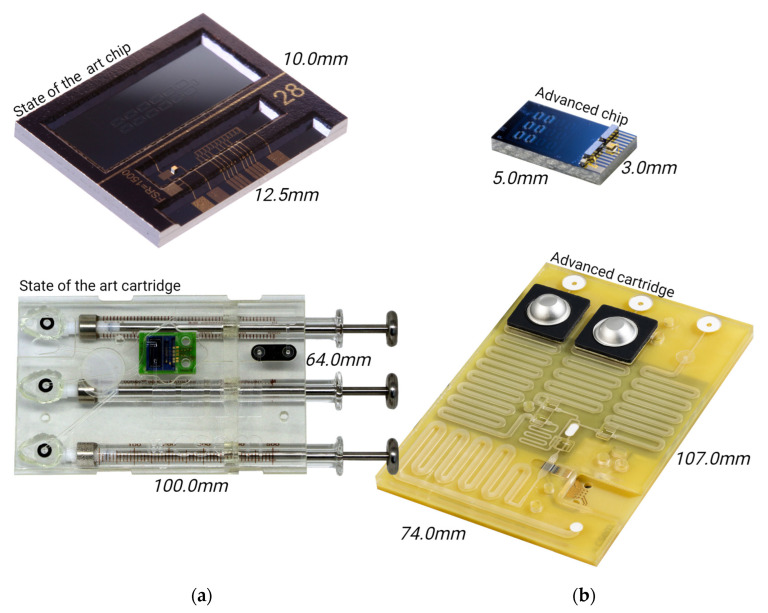
Comparison of the state-of-the-art chip and cartridge ((**a**) [18]) versus the advanced chip and advanced cartridge (**b**) (present study).

In this work, we present several significant advances over the state of the art as presented in [18] (see also Figure 1):

- Miniaturization of the hybrid biosensor PIC;
- Development of wafer-level processes for hybrid integration of the light source, detector, and temperature sensor on the PIC;
- Development of wafer-level processes for material-selective chemical and biological surface modification of the PIC;
- Scalable processes for integration of the PIC in a microfluidic cartridge;
- Successful biosensing experiments, detecting pathogen-specific DNA using hybrid PICs.

Importantly, these processes were not only developed and optimized as such, but they were designed to be mutually compatible and constitute a coherent workflow for manufacturing functional microfluidic cartridges with hybrid PICs, which can be used in combination with a prototype readout instrument.

In parallel to the development of the hybrid PIC biosensor platform, quantitative Polymerase Chain Reaction (qPCR) tests for detection of three aquaculture pathogens (*Aeromonas salmonicida* (A. sal) [22], *Vagococcus salmoninarum* (V. sal) [23], and *Yersinia ruckeri* (Y. ruc) [24]) were developed. The pathogen-specific DNA sequences used for developing the primer-probe sets of these assays can also be used as the starting point for the development of PIC-based pathogen detection assays. To demonstrate the functionality of the integrated biosensor system, PICs were biofunctionalized with capture probes for A. sal and used for detection of pathogen-specific DNA.

## 2. Materials and Methods

### 2.1. Materials

DNA probes were ordered at Integrated DNA Technologies, Inc. (IDT, Coralville, IA, USA). All other chemicals were purchased at Sigma-Aldrich (St. Louis, MI, USA) or VWR International (Radnor, PA, USA) and were used without further purification, unless specified otherwise.

### 2.2. TriPleX^TM^ PIC

The integrated photonic biosensing platform uses TriPleX^TM^ waveguide technology [25,26]. PICs were fabricated on 4″ Si wafers (thickness 525 µm) by Lionix International (Enschede, The Netherlands), based on a single-layer waveguide core of stoichiometric silicon nitride (Si_3_N_4_), sandwiched between a 6 µm thermal silicon oxide (SiO_2_) bottom cladding and a 4 µm SiO_2_ top cladding. The waveguides have a height of 100 nm and a width of 1000 nm. The choice of stoichiometric silicon nitride as the waveguide core material in the TriPleX™ PIC enhances the performance of the photonic biosensing platform by enabling efficient light confinement, low optical loss, and seamless integration with CMOS processes, ultimately leading to improved sensitivity, signal integrity, and overall functionality of the biosensing system.

The general features of the PIC design were based on those used in previous work [18]. Light is coupled in and out of the PIC via gratings. Each PIC contains 1 double-port input grating and 8 single-port output gratings. The on-chip waveguide circuitry comprises Y splitters and combiners for connecting the in- and output gratings to 6 aMZI biosensor elements and 2 auxiliary structures, which are used for linearization and calibration purposes. Furthermore, Cr-Au electrodes and contact pads for hybrid integration and wire bonding were patterned on the PIC by lift-off photolithography.

Each aMZI biosensor consisted of two spiral sensor arms (a signal arm and a reference arm) arranged in a stadium (arena) shape with a geometric path length of 12.5 mm. Two different spiral designs were used, a ‘small spiral’ with a footprint of 350 × 400 µm, and a ‘large spiral’ with a footprint of 400 × 650 µm. One of the arms had an additional path length of 680 µm (outside the spiral region). Because of this asymmetry, the transmission spectrum of the aMZI had a sinusoidal shape [15,16].

For each sensor element, the SiO_2_ top cladding was etched away locally at the position of one or both of the waveguide spirals, resulting in so-called ‘sensing windows’ [17]. In the sensing window, the light propagating through the waveguide can interact with the sample via its evanescent field, and the waveguide is accessible for biofunctionalization, enabling the aMZI to function as a biosensor. A change of the effective refractive index of the solution above the sensing window causes a change of the optical path length, which in turn results in a phase shift of the transmission spectrum. Such a refractive index change may be caused by a change in the bulk refractive index of the solution but also by the binding of specific biomolecules to immobilized receptors on the waveguide [27].

To monitor the phase shift of the transmission spectrum during measurement, the input wavelength is continuously varied over a range of approximately 3 nm at a frequency of 10 Hz, and the resulting transmission spectrum for each wavelength scan is recorded. The response to refractive index changes can be expressed as a shift of the spectrum in nm (or pm) on the wavelength axis. Note that the refractive index sensitivity depends on the geometric path length and the asymmetry of the arms, which can be adjusted in the aMZI design. This tunability is one of the main benefits of aMZI biosensors over other PIC designs such as microring resonators. For the current aMZI design at the operating wavelength of approximately 850 nm, the theoretical sensitivity to changes in bulk refractive index is approximately 1900 nm per refractive index unit (RIU).

### 2.3. Hybrid Integration

Flip-chip bonding is a straightforward method for directly integrating top-emitting optical sources, such as Vertical Cavity Surface Emitting Lasers (VCSELs), and top-illuminated photodiodes (PDs) onto a substrate. This is because the electrical connections and optically active areas are both placed on the top surface of the chip. The uppermost layer of electrical interconnects terminates on metal pads. Moreover, bonding optoelectronic components requires submicron alignment precision, which is achieved using machine vision of alignment fiducial marks realized on both the III-V dies and the PIC substrate.

Die-level and wafer-scale processes for flip-chip bonding were developed and validated using a Fineplacer Femto 2 die bonder (Finetech GmbH, Berlin, Germany) equipped with a conductive global heating system and a laser-assisted local heating system. Furthermore, the system is equipped with wafer handling, automated dispensing (to underfill the flip-chip bonded dies), and tool changing modules, enabling development of high-volume hybrid integration processes.

For die-level bonding processes, conductive heating can be used to heat both the substrate holder and the tool head. However, this technique is not suitable for wafer=level bonding because the entire wafer would need to be (re)heated each time a component is placed, which may result in release or displacement of previously bonded components. Consequently, laser-assisted heating was adopted for wafer-scale bonding. This method uses laser power that has a Gaussian-shaped beam spot, 0.6 mm in diameter, to locally heat and reflow the solder pads of components on a specific area of the wafer by applying it to the back of the wafer. This approach allows successive components to be transferred onto the wafer without compromising the bonds already made.

The following components were integrated on the PIC:

- Single-mode 850 nm polarization stable VCSEL (Trumpf Photonic Components GmbH, Ulm, Germany);
- 1 × 4 arrays of GaAs PIN PDs (Trumpf Photonic Components GmbH, Ulm, Germany);
- Negative Temperature Coefficient (NTC) thermistor (VH05, Mitsubishi Materials Corporation, Tokyo, Japan).

After bonding of these components, a fully automatic wire bonder is utilized to interconnect the N-side of the VCSEL and the unbonded side of the NTC to the PIC.

### 2.4. PIC Biofunctionalization

Prior to immobilization of bioreceptors, a material-selective surface modification process was applied [17]. This process results in a carboxylic acid terminated layer on the exposed Si_3_N_4_ waveguide in the sensing window, while the surrounding SiO_2_ surface is modified with a poly(ethylene) glycol (PEG)-based antifouling layer. The carboxylic acid groups can be used for further reaction and immobilization of bioreceptors, while the PEG layer prevents non-specific adsorption of biomolecules. It has been shown previously that this approach confines analyte binding to the waveguide surface, which results in a higher sensitivity and improved limit of detection [28]. Coated PICs were stored in a glovebox, cleaned by sonication in ultrapure water and methanol, and dried in a stream of nitrogen before further use. Coatings were characterized by measurement of the water contact angle (WCA) and X-ray Photoelectron Spectroscopy (XPS) on dedicated test areas on the wafer.

Chemical activation of the carboxylate coating on the waveguides was performed by dispensing a 60 µL drop of a freshly prepared solution of 0.2 M EDC (1-ethyl-3-(3-dimethylaminopropyl)carbodiimide hydrochloride) and 0.1 M NHS (N-hydroxysuccinimide) in 10 mM buffer solution of MES (2-(N-morpholino)ethanesulfonic acid) of pH 5.5 and incubating it for 15 min. Biofunctionalization of waveguides in individual sensing wells was done using an automated piezo-driven, non-contact dispensing system (SciFLEXARRAYER S3, Scienion, Berlin, Germany) equipped with a PDC80 nozzle. A spotting solution was prepared based on a protocol from the literature [29], optimized for spotting on the aMZI PICs. To 18 µL of this solution, 2 µL of a 100 µM DNA solution (capture probe or control probe) was added, followed by vortex mixing and centrifugation. A total of 6.75 nL of DNA solution was dispensed in each sensing well. On each PIC, 3 sensing wells were spotted with the capture probe and 3 sensing wells were spotted with the control probe. Probe sequences had a terminal amine group on their 5′ end for reaction with the EDC/NHS-activated carboxylic acid groups on the waveguide surface. A triethylene glycol spacer (iSp9) was added between the terminal amine group and the nucleotide sequence. After spotting, PICs were washed 20× with saline sodium citrate (SSC) buffer, 10 mM PBS (140 mM NaCl), and ultrapure water.

### 2.5. Microfluidic Cartridge

The microfluidic cartridge assembly is based on a sandwich design, where the bottom of the cartridge is formed by a customized 2.3 mm thick two-layer FR4 Printed Circuit Board (PCB) with Electroless Nickel and Palladium Immersion Gold (ENEPIG) coating to enable wire bonding with gold. PCBs were supplied by DB Electronic (Baden, Switzerland). The fluidic top part (‘lid’) is milled out of 3 mm thick high melting Cyclic Olefin Copolymer (COC), provided by Microfluidic Chipshop (Jena, Germany).

#### 2.5.1. Cartridge Coating

Microfluidic cartridge parts (PCB and lid) were provided with a hydrophilic, anti-biofouling PEG coating to enhance liquid flow and prevent unwanted adsorption of biomolecules to the channel walls. PCBs were coated on one side; lids were coated on both sides. First, parts were cleaned by ultrasonication in acetone and exposure to a 0.2 mbar air plasma (Atto plasma generator, Diener Electronic, Ebhausen, Germany). A proprietary coating formulation comprising a surface-reactive PEG compound was prepared. A uniform layer was deposited on the parts using an ultrasonic spray coater equipped with a Focusmist nozzle (ND SP, Nadetech, Navarra, Spain) operated at a power of 3 W. The nozzle-to-sample distance was 54 mm, and a nozzle speed of 2 m/min was used at a flow rate of 10 mL/h. Spray coating was followed by curing using a UV crosslinker (CL-508, Cleaver Scientific, Rugby, UK) equipped with 312 nm UV sources while purging with argon gas. Finally, substrates were cleaned by ultrasonication in ultrapure water and isopropyl alcohol. Coatings were characterized by measurement of static and advancing WCAs.

#### 2.5.2. PCB Assembly

A Zener diode (GDZ5V6LP3-7, Diodes, Inc., Plano, TX, USA) was soldered in a reflow oven with soldering paste between the connection leads of the VCSEL to prevent damage to the VCSEL by electrostatic discharge (ESD). The Zener diode integration was done prior to the anti-fouling coating of the PCB due to temperature restriction.

In clean room conditions (ISO class 7), the hybrid PIC was placed into the cavity of the PCB with electrically conductive glue to establish the contact between the two connections for the backside heater. In the next step, the hybrid PIC was encapsulated flush with the PCB surface, using a protective mask for the biofunctionalization layer during UV curing. In a following step, ball-to-wedge Au wire bonds (25 µm diameter Formax, Heraeus Electronics, Hanau, Germany) were formed between the hybrid PIC and the PCB with a wire bonder (Fineplacer PICO MA, Finetech GmbH, Berlin, Germany).

Before and after each of the hybrid PIC integration steps, the VCSEL and PDs were tested for their proper functioning by using probes directly pressed onto the bond pads of the PIC. The positioning of the PIC in the cavity was evaluated using a Keyence VK-X110 Laser Scanning Microscope (LSM) (Keyence, Osaka, Japan).

#### 2.5.3. Lid Assembly

Laser cut degassing and liquid membranes (Emflon PTFE, 1.0 µm pore size, Pall, Port Washington, NY, USA) were glued, and a pierceable septum (MVQ, thickness 0.5 mm, Shore A hardness 40, Kubo Tech AG, Illnau-Effretikon, Switzerland) was tapped into the designated cavities.

The blister seats were fabricated using a Prusa SLS1 3D printer and Prusament resin of type Tough Anthracite Grey (Prusa Research a.s., Prague, Czech Republic). Blisters were purchased from Microfluidic Chipshop (Jena, Germany) and custom-filled with buffers for the different experiments. The cartridge is designed such that one blister seat works with blister volumes up to 500 µL (Fluidic 1175), while the second blister seat is designed for blister volumes up to 350 µL (Fluidic 1182).

The sample port is optimized to work with a 0.3 mL insulin syringe (Micro-fine U100, BD, Franklin Lakes, NJ, USA). The sample size can be up to 300 µL. The instrument actuation compensates for lower volumes, e.g., if the buffer blisters are smaller than the maximum, if they do not release their full volume, or if less sample is injected by the user, the process can be adapted accordingly due to the liquid level check windows in the cartridge and flow front sensors in the instrument.

After the lid is flipped, a double adhesive tape (CSEM D150) was applied and compressed for 60 s with a force of 4 kN to activate the tape. For the final assembly, the lid with removed liner was placed into an assembly station where the PCB was put on top. Again, a compression step of 60 s and 4 kN was applied.

#### 2.5.4. Cartridge Testing

For testing leak tightness, degassing, septum piercing, and pumping of driving fluids, a simplified cartridge was used, where the cartridge’s liquid interface was connected to a peristaltic pump (Ismatec ISM597D, Glattburg, Switzerland) that transported dyed (food colorant) ultrapure water. Additionally, for the tightness tests, the blister seats were sealed, and a pressure sensor was attached after the liquid stop membrane. For testing the driving fluid and the pierceable membrane, a simplified cartridge was used in which only a taped septum and an intermediate meander was connected to the peristaltic pump. Dispensing tests were performed with a syringe (BBraun Injekt 10 mL, 4606108V, Melsungen, Germany), which was partially filled with water and connected via Y-connection to a 3D-printed blister seat and a flow sensor (Fluigent Flow Unit, type L, Le Kremlin-Bicêtre, France).

#### 2.5.5. Bulk Refractive Index Change Testing

Cartridges were prepared with PICs with balanced aMZIs, which were used without surface functionalization, and two blisters containing a standard PBS buffer solution. As the sample, a PBS buffer solution with a 40 mM lower salt (NaCl) concentration was used. To the content of all blisters, 0.5% (*v*/*v*) ProClin 300 was added as a preservative to prevent microbial growth during blister storage. First, the content of the first blister was pumped over the sensor to generate a baseline signal. Then, the sample was pumped over the sensor. The 40 mM concentration change resulted in a bulk refractive index change of the solution of approximately 4 × 10^−4^ RIU, which should result in a clearly measurable phase shift of the aMZI output signal. Finally, the content of the second blister was pumped over the sensor. The refractive index of the solution returned to its original value, and therefore the sensor signal was also expected to return to the baseline.

### 2.6. Readout Instrument

The readout instrument controls the cartridge processing with minimal user interaction. The main functions are liquid handling, PIC control, and data analysis. Liquid handling includes pressing the blisters with the buffers, supporting sample application, priming, and driving the liquids (buffers and sample) over the PIC with a well-defined constant flow rate. For PIC control, electrical contacts to the cartridge must be established. The instrument electronics drive the VCSEL with a defined current profile and read out the PDs in parallel. The recorded signals must be analyzed during the measurement to track the phase shift caused by changes of the effective refractive index near the PIC surface. Electronics and software concepts for VCSEL driving, PD readout, and acquisition and processing of raw sensor data to obtain a sensorgram (i.e., a graph of phase shift vs. time) were re-used from a previously developed instrument [18]. Temperature control of the PIC is required for stabilizing the emission spectrum of the VCSEL.

### 2.7. Pathogen Biomarker Discovery

A comprehensive pilot study was carried out to identify the most important pathogens for European salmon aquaculture. Relevant salmon pathogens were selected according to their commercial importance for aquaculture, their safety/risk group, the availability of pure reference strains, and their published DNA sequences [30].

Based on this study, three pathogens were selected for assay development (Table 1). All pathogens were ordered via DSMZ (Deutsche Sammlung von Mikroorganismen und Zellkulturen) and were cultivated under the recommended conditions. Growing cultures were subsequently used to prepare pure reference DNA extracts and, after plating on nutrient agar plates, to calculate the number of Colony Forming Units (CFUs) per milliliter medium (CFU/mL).

Primer–probe sets for the selected pathogens were designed after sequence alignments with sequences obtained from the NCBI database (http://www.ncbi.nlm.nih.gov/, accessed on 26 April 2021) and using PrimeQuest™ software (Version 2.2.3, Integrated DNA Technologies, Coralville, IA, USA). Double-quenched probes were used with 6-FAM, SUN, and HEX as 5′ reporter dyes in combination with an internal (Zen) and 3′ (Iowa Black, IABkFQ) fluorescence quencher.

The phenylalanyl-tRNA synthetase gene (*pheS*) for V. sal [31], the glutamine synthetase gene (*glnA*) for Y. ruc [32], and the virulence array protein gene (*vapA*) for A. sal [33,34] were chosen as targets for qPCR, and for the latter target also for subsequent detection on the PIC. In the following, the focus is only on the assay development and measurement of the pathogen A. sal, which was also selected as the target for the PIC assay.

### 2.8. Pathogen DNA Sample Preparation

Bacterial DNA from cell pellets and colony picks, as well as environmental DNA (eDNA) from spiked water samples with bacterial concentrations between 1 and 100 million CFU/mL, filtered by cellulose acetate filters (to mimic sampling of water at aquaculture facilities) were extracted using the Chelex-100 method [35]. Cell pellets and colony picks were mixed with 98 µL of a 10% Chelex suspension (10 g Chelex-100 added to 90 mL Tris-EDTA (TE) buffer) and 2 µL proteinase K (20 mg/mL) in a 1.5 mL reaction tube. Cellulose acetate filters were cut into small pieces, placed in 2 mL reaction tubes, and topped up with 990 µL 10% Chelex suspension and 10 µL proteinase K. Samples were placed in a ThermoMixer (Eppendorf, Hamburg, Germany) at 56 °C and 1300 rpm for 30 min with brief vortexing after 15 min followed by 20 min incubation at 99 °C. The samples were then centrifuged in a tabletop centrifuge at 15,000 rpm (SelectSpin 21, Select BioProducts, Edison, NJ, USA), and the DNA-containing supernatant was transferred to a clean reaction tube for later use. Filtration experiments were conducted by filtering one liter of ultrapure water spiked with frozen media (CFU equivalents of 10 to 1,000,000) through cellulose acetate filters (pore size 0.45 μm, filter diameter 47 mm, Pieper Filter GmbH, Bad Zwischenahn, Germany) via a vacuum pump. The effects of time, temperature, and storage with or without ethanol on the DNA recovery rate were determined after 1, 3, 7, 10, and 14 days.

### 2.9. qPCR Assay

PCR reaction mixtures were prepared comprising 5 μL ProbeMasterMix (Genaxxon, Ulm, Germany), primers and probes at 500 nM and 250 nM, respectively (PrimeTime qPCR assay, IDT), and 1 μL of template DNA in 10 μL total reaction volume. The reactions were cycled in a Real-Time PCR Detection System (CFX96 Touch, Bio-Rad Laboratories, Hercules, CA, USA) with the following PCR program: polymerase activation at 95 °C for 15 min, followed by 40 cycles of 95 °C for 10 s and 60 °C for 10 s. For A. sal, a synthetic, 208 bp long gene fragment (gBlocks™, IDT), which contained the 91 base pair (bp) long PCR product surrounded by 58 and 59 protective bases, acted as a quantification standard for all samples. The subsequent software-supported evaluation, visualization, and quantification were carried out with Bio-Rad CFX Maestro 2.3 software (version 5.3).

To test the robustness of the qPCR assays as a reference test for the PIC analyses, the DNA recovery rates were calculated after filtration of media with a known CFU count and DNA extraction from cellulose acetate filters. No-template and negative controls were checked for all qPCR assays and were part of each individual qPCR analysis.

### 2.10. Pathogen DNA Detection with Hybrid PIC System

For these experiments, PICs with unbalanced aMZIs were material-selectively coated. Then, 3 detection aMZIs on a PIC were biofunctionalized by covalently immobilizing the complementary DNA sequence (capture probe) on the surface of the waveguide. For the other 3 aMZIs, a non-complementary DNA sequence (control probe) was covalently immobilized as a negative control. The measurement procedure is the same as for the bulk refractive index measurements described above, but different buffer and sample solutions are used.

For detection of the aquaculture pathogen A. sal, the target sequence and the nucleotide sequence for the capture probe are available based on the qPCR assay and are shown in Table 2. As the control, the non-complementary probe CT47 was used [36].

When the PIC is exposed to a sample containing the target DNA sequence, both the detection and the control aMZIs will respond to a bulk change in the refractive index of the solution, but only the detection aMZIs will bind the target DNA. Therefore, it is useful to look at the difference between the detection and control signals, which corresponds to the net result of the target DNA binding to the sensor surface.

## 3. Results and Discussion

### 3.1. Design and Development

#### 3.1.1. Hybrid PIC Design

Compared to the hybrid PICs that were previously used [18], several improvements were implemented to improve the manufacturability and reduce the cost without compromising the sensitivity and the number of sensors per chip. By optimizing the design and reducing the spacing of the sensing windows, the footprint of the biosensing area of the PIC (which has to be in contact with the sample) was reduced.

**Figure 2:**
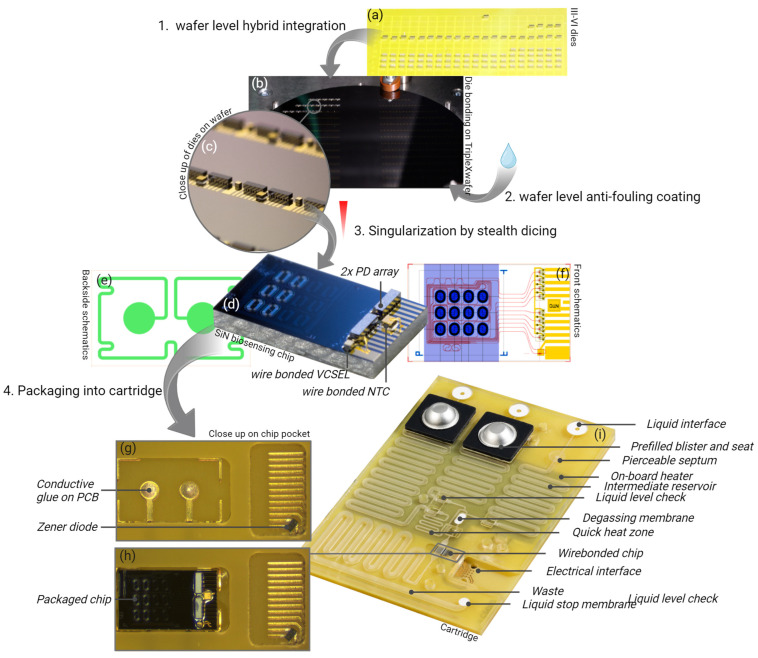
(**a**,**b**) Wafer level flip-chip bonding of PDs. GaAs PD dies are individually transferred from a carrier and bonded to a wafer by laser-assisted local reflow soldering (right). (**c**) Close-up on multiple VCSELs, PD arrays, and NTCs populated on a wafer. (**d**) Completely assembled, wire-bonded and stealth-diced hybrid PIC (with ‘large’ spirals), VCSEL, PD arrays, and NTC. (**e**,**f**) Design of the hybrid PIC. (**f**) Front side with photonic waveguide circuitry (red), Cr-Au metallization (yellow), etched sensing windows (blue) with ‘small’ spirals, and microfluidic flow channel (purple). (**e**) Backside with Ta-Pt resistive heater. (**g**,**h**) Close-up of the PIC cavity of the PCB. (**a**) After dispensing electrically conductive glue; (**b**) after placement and encapsulation of the hybrid PIC. (**i**) Photograph of the microfluidic cartridge. The labelled features of the cartridge are discussed in the text.

The design of the PIC is shown in Figure 2f. The outer dimensions are 3.0 × 5.0 mm^2^, which is an 8-fold reduction compared to the footprint of the previous PIC (120 mm^2^) [18]. Compared to the previous design, the layout of the 12 aMZI waveguide spirals was changed from a 2 × 6 to a 4 × 3 array, and the input and output waveguides were re-routed accordingly. The liquid flow direction is over the width of the chip (the flow path is indicated by the purple shaded area), reducing the contact area between the chip and the microfluidic cartridge.

The two waveguide spirals that make up an aMZI sensor can be in a ‘balanced’ or an ‘unbalanced’ configuration [17]. In the balanced configuration, both spirals are exposed to the sample solution (as shown in Figure 2f). In the unbalanced configuration, only one of the spirals is exposed to the sample solution, while for the other spiral the sensing window is not etched open, so it remains buried under the oxide layer. Both configurations are useful for assay development and system validation. Therefore, PICs with either balanced or unbalanced aMZIs were fabricated.

For hybrid integration of components, an area of 1.5 × 3.0 mm^2^ is designated on the PIC. This area contains the double-port input grating, on top of which the VCSEL will be bonded. Light emitted by the VCSEL is coupled into two waveguides, which are both split into four channels by subsequent Y splitters, thus creating eight waveguide channels (six aMZI biosensor channels and two auxiliary channels). Light from each channel is coupled out of the PIC by a one-port grating. Two 1 × 4 PD arrays are bonded on top of the eight output gratings to detect the intensity of the output light from each channel. In addition to the active optical components, an NTC thermistor is integrated on the PIC to monitor the temperature. The positions and dimensions of the bond pads for contacting VCSEL, PDs, and NTC were designed to match the anode and cathode of the components. For the eight photodiodes, a common anode is used. Metal (Au-Cr) tracks run from all anodes and cathodes to the edge of the PIC, enabling wire bonding from the PIC to the PCB.

On the backside of the PIC (Figure 2e), a resistive heater is patterned by depositing a meandering Ta-Pt line with a width of 100 µm and a length of 20 mm and circular contact pads with a diameter of 1 mm, resulting in a nominal resistance of approximately 600 Ohm.

#### 3.1.2. Integration of Components on Hybrid PIC

A complete process for wafer-level production of hybrid PICs was developed, in which four components (one VCSEL, two PD arrays, and one NTC thermistor) are populated onto every die on the PIC wafer. By choosing different bonding technologies and temperatures for the different components, and by applying them in order of decreasing temperature, it is ensured that the processes are mutually compatible, and previously bonded components are not released or moved during subsequent steps. The VCSELs are populated first via Au-Au thermocompression bonding at 330 °C while applying a force of 1.5 N. Next, PD arrays are bonded using solder reflow at 300 °C (shown in Figure 2b). Finally, the NTC thermistors are bonded at 150 °C using electrically conductive adhesive and are post-baked at 90 °C.

By leveraging advanced pick-and-place tools and laser heating techniques, a component can be placed and bonded in 30 s with a precision better than 300 nm. This efficiency enables the population of 120 photonic components onto a wafer per hour, equating to the assembly of 30 complete hybrid PICs, as illustrated by Figure 2c. This exemplifies the effectiveness of wafer-scale hybrid PIC integration for volume production and substantially reduces intensive labor work.

A wedge-wedge wire bond process is employed at 110 °C to establish the interconnection between the N-side of the VCSEL and the PIC, as well as the unbonded side of the NTC to the PIC. Following this step, the flip-chipped bonded PD arrays and VCSEL undergo underfilling with index-matching optical glue. This underfilling process is carried out using an automated dispenser and is followed by curing at 100 °C. A completely assembled hybrid PIC is shown in Figure 2d, with the bonded VCSEL, PDs, and NTC.

#### 3.1.3. PIC Surface Modification

The hybrid integration processes described above require high temperatures, which are not compatible with the presence of organic coatings on the wafer. Therefore, surface modification of the PIC has to take place after the integration of active components.

**Figure 3:**
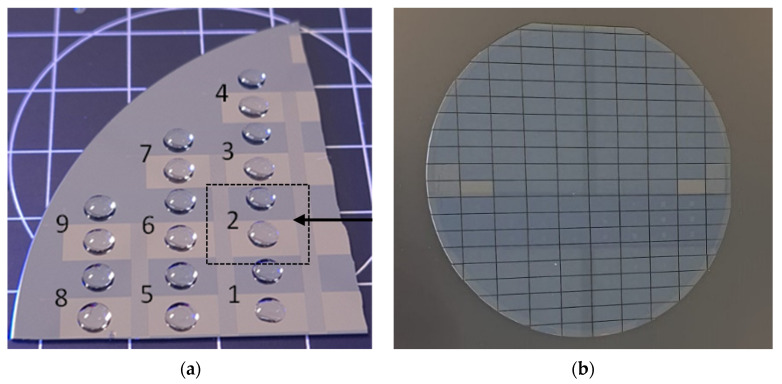
(**a**) Part of a test wafer with large Si_3_N_4_ and SiO_2_ areas (numbers indicate the areas of separation) for characterization of coatings by WCA measurements. (**b**) Coated wafer after singulation by stealth dicing.

The material-selective surface modification process was previously used for coating PICs at the chip level [17]. However, for a scalable manufacturing process, coating should be done at the wafer scale. Therefore, the process was scaled up, and it was demonstrated that it is suitable for wafer-scale surface modification. To facilitate process development and characterization of the coatings, wafers (4″) with large (5 × 10 mm^2^) areas of Si_3_N_4_ and SiO_2_ were used. Figure 3a shows part of such a test wafer after deposition of 3 µL droplets of ultrapure water, which were used for measuring the WCA. The average WCA value for the COOH-terminated coating on Si_3_N_4_ was 35 ± 1°, and the WCA for the PEG coating on SiO_2_ was 46 ± 1°. These WCA values are identical to those previously measured on individual chips, thus indicating that the coating process can successfully be carried out at the wafer scale.

Coated wafers were then singulated by stealth dicing (Figure 3b) [37]. This process was carried out by DISCO HI-TEC Europe (Munich, Germany). Stealth dicing uses laser pulses to create sub-surface defects while keeping the top and bottom surface of the wafer intact. It is a mild, low-temperature process that does not require cooling water and does not produce debris. For the dicing of wafers with sensitive surface structures and properties, stealth dicing technology offers several important advantages over alternative methods such as traditional blade dicing (using cooling water to remove debris, which may affect the coatings) and ablative laser dicing (which involves significant heat and debris production). WCA values were measured before and after stealth dicing, and the results were identical to those of chips that were coated after dicing, thus indicating that stealth dicing does not damage the coatings.

Next, the material-selective coating process was applied to chips with integrated components. The current-voltage-light (I-V-L) characteristics of VCSEL and PDs were measured before and after coating. The results indicate that the functionality of the components is not compromised by the coating process.

Because of the mild conditions of the stealth dicing process, it is compatible with the presence of integrated components and coatings on the wafer. At the same time, the presence of these components and metal contact pads on the wafer does not hamper the stealth dicing process, as long as sufficient empty space is available around the dicing lines. The dicing street should have a minimum width of 10% of the wafer thickness, in this case corresponding to approximately 60 µm, i.e., 30 µm on both sides. This was taken into account in the PIC design.

Thus, the developed processes for flip-chip hybrid integration, material-selective surface modification, and stealth dicing constitute a scalable process flow and enable wafer-scale fabrication of hybrid biosensor PICs.

#### 3.1.4. Microfluidic Cartridge

The fully assembled microfluidic cartridge is shown in Figure 2e. The outer dimensions (length × width × height) of the cartridge without the blister seats and blisters are 106 × 74 × 5 mm^3^.

Below, the main functions, design features, and verification results of the cartridge are described with reference to the labelled items as shown in Figure 2e.

**Figure 4:**
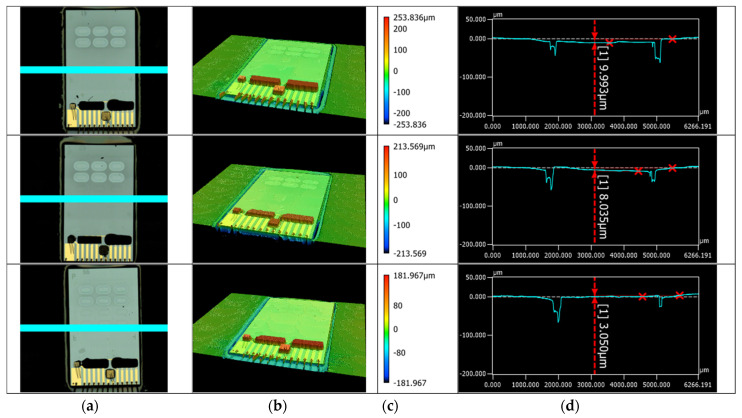
LSM measurement of 3 hybrid PICs integrated in the PCB. (**a**) Optical microscopy image; (**b**) 3D LSM height image; (**c**) scale bar for the height; (**d**) surface profile measurement on the cross-section indicated with turquoise line in (**a**). The height difference between the PCB and PIC surface varies between 3 and 10 μm. Measurement errors were observed at the transition between PCB and PIC, where the transparent glue cannot be well resolved.

- Liquid interface—connects to the liquid actuation ports of the instrument, allowing driving fluid to be pumped into the cartridge.
- Blister seats with pre-filled blisters with run buffers and (optional) reagents—connects to the blister squeezers of the instrument. For prototyping, the cartridge lid was fabricated by milling, and the blister seats were 3D printed. For volume production, the lid can be injection molded with the blister seats integrated in the injection molded part, thus reducing the number of parts and assembly steps.
- Pierceable septum—connects to the sample application port of the instrument, through which the user injects the sample using a syringe. After removing the syringe needle, the septum seals the inlet, allowing the sample to be transported to the PIC by the driving fluid. To ensure the proper functioning of the pierceable septum, it was tested on its tightness after being punctured with the syringe needle. After being pierced once, the taped septum can withstand more than 1000 mbar overpressure in the channels.
- Intermediate reservoirs—meandering channels used for temporarily storing and pre-heating the sample (injected by the user) and buffer solutions (from the blisters) before being transported over the PIC by the driving fluid.
- Waste reservoir and liquid stop membrane—after moving liquids over the PIC, all waste is stored on the cartridge. To accommodate for the required volume, this meandering channel has a larger cross section than the other channels. A gas-permeable liquid stop membrane prevents spillage of liquids into the instrument in the case that the waste reservoir is completely filled.
- On-board heater and quick heat zone—using a resistive heater integrated on the PCB, all liquids can be brought to the required temperature before being transported over the PIC.
- Liquid level check—to compensate for differences in sample and blister (filling and recovery) volumes, all liquids are first transported to the end of their respective intermediate reservoir to reach a well-defined ‘starting position’ for being transported over the PIC. When the flow front reaches a predefined position, this is detected by a flow front sensor in the instrument, and the pumping is stopped. When all liquids have reached their starting position, the first buffer is pumped over the PIC, and data acquisition is started.
- Degassing membrane—because the aMZI biosensors detect changes in the refractive index, the presence of air bubbles on the PIC is detrimental for sensor performance and has to be avoided. To prevent gas bubbles from reaching the PIC, the liquids pass a PTFE (polytetrafluoroethylene) degassing membrane just before reaching the PIC, allowing gas to escape. Tests showed that the glued membrane withstands more than 1000 mbar overpressure without losing its function.
- Electrical interface—the 4 × 4 array of contact pads connects with the pogo pins of the spring-loaded electrical connector unit of the instrument. The contacts are used for driving the VCSEL and PIC backside heater and for reading out the photodiodes and NTC thermistor. Two additional contact pads are positioned nearby for driving the on-board PCB heater.
- Hybrid PIC—Figure 2g shows the PIC cavity in the PCB after deposition of electrically conductive glue on the contact pads for the PIC backside heater. Figure 2h shows the situation after placement of the PIC and dispensing and curing of the encapsulation glue. The PIC integration process was optimized such that the PIC surface was flush with the PCB surface, with a height difference between 3 and 10 μm as shown in Figure 4. The precise alignment of the top surfaces of the PIC and the PCB allowed leak-tight sealing of the cartridge by double adhesive tape. Pressure tests of the assembly with a flow rate of 100 μL/min reached burst pressures of at least 500 mbar. In the final step of PCB assembly, wire bond connections were made between the contact pads on the PIC and the contact pads on the PCB.

**Figure 5:**
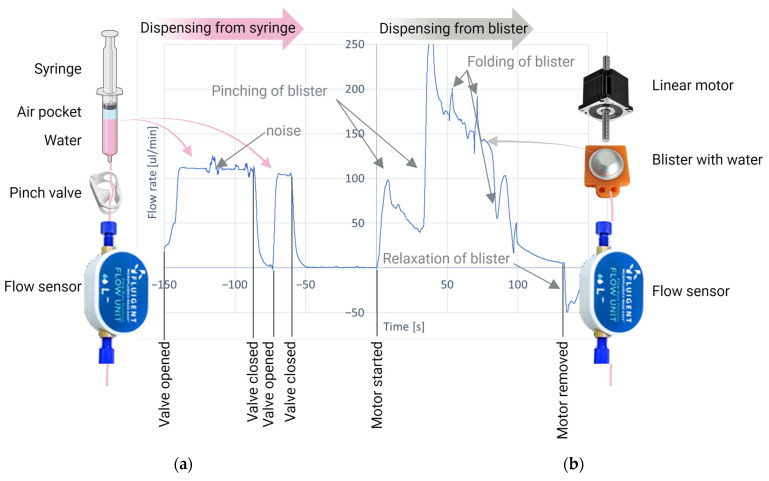
Comparison between flow rate control by direct dispensing from (**a**) syringe; (**b**) blister. The flow rate was measured using the same flow sensor in both situations. The magnitude of signal noise can be observed during the dispensing from the syringe, During the dispensing from the blister, the first flow rate increases when the blister is punched by the 3 needles in the blister seat. Later, the flow rate reduces and starts to pulsate when the blister starts to fold.

In the previously developed cartridge [18], pre-filled syringe-like structures were used to store liquid buffers and reagents. By pushing the syringe plunger, a well-controlled flow rate can be achieved (Figure 5a), which is important for PIC signal stability. However, it was found that the evaporation rate of aqueous liquids from the prefilled syringes was significant, even when a sacrificial seal was added to minimize diffusion. Therefore, an alternative solution for storing and transporting liquids based on blister pouches was developed for the current cartridge.

The use of blister pouches is a well-known solution for reagent storage in microfluidic cartridges [38]. Blister materials are optimized for their barrier properties and can have an evaporation rate <1% per year. When emptying a blister, in principle its contents can be directly transported over the PIC, so no additional liquid-driving mechanism would be needed. However, dispensing tests (Figure 5b) revealed that directly emptying a blister (using a linear stepper motor at a constant rotation speed) results in a strongly varying flow rate. This is related to the dome shape of the blister and the unpredictable way the blister folds and wrinkles during compression. Therefore, it was decided to first release the content of the blisters into an intermediate reservoir in an automated way (due to higher overall volume reproducibility than in a manual way), as shown in Figure 2e. From there, the liquids can be transported further at a well-controlled flow rate using a driving fluid.

The best flow control can be achieved by using a (non-compressible) fluid to drive the sample and buffers over the PIC. A first test was conducted, where water was used as the driving fluid. To reduce mixing of the driving fluid and the (aqueous) sample or buffer, an additional air bubble was deliberately added to separate the different liquid plugs. Note that the air bubble is removed by the degassing membrane before reaching the PIC and therefore does not interfere with the measurement.

**Figure 6:**
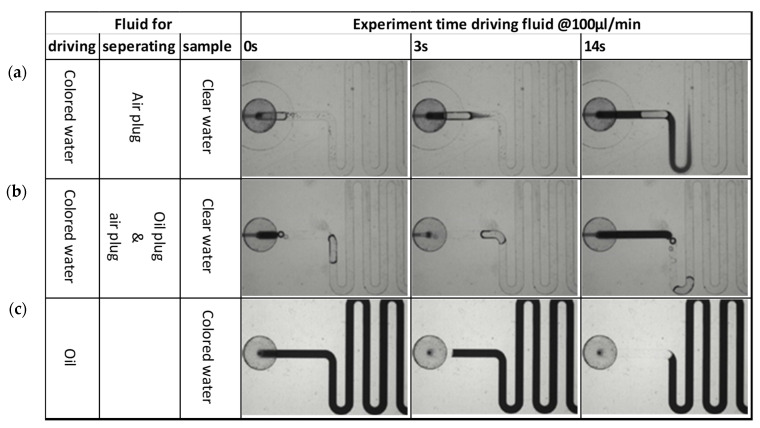
Driving aqueous solutions in a channel with rectangular cross-section using different driving fluids: (**a**) water, using air to separate liquid plugs; (**b**) water, using oil and air to separate liquid plugs; (**c**) oil.

However, it was observed (Figure 6a) that despite the presence of the air bubble, significant mixing between the two liquid plugs (clear water and colored water) takes place in a few seconds due to Taylor dispersion [39]. Clearly, separation of two aqueous liquid plugs by an air bubble is not effective. Mixing was reduced when an additional oil plug was used for separation (Figure 6b). However, implementing this solution would increase the complexity of the instrument, which would have to supply both air and oil to the cartridge. Therefore, it was decided to use oil as the driving fluid (Figure 6c). In this way, mixing between the driving fluid and sample or buffer is effectively prevented, and only one driving fluid is required.

#### 3.1.5. Readout Instrument

**Figure 7:**
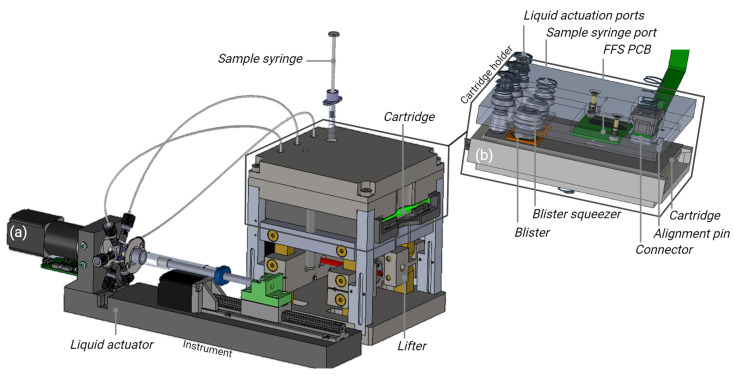
Design of the prototype readout instrument. (**a**) Basic functional units; (**b**) interfaces to the microfluidic cartridge.

A compact automated instrument was designed for cartridge handling, signal acquisition, and data analysis (Figure 7).

After insertion, the cartridge is raised by a scissor lifter. While the cartridge is lifted, the blisters are pushed against spring loaded blister squeezers, releasing the buffers into the intermediate storage channels of the cartridge (cf. Figure 2i).

Further interfaces to the cartridge are attached from above; see Figure 7b. Spring-loaded liquid actuation ports ensure tight contact between the cartridge and liquid handling system of the instrument. The liquid actuator (Figure 7a) consists of a glass syringe, a linear stepper motor drive, and a rotary valve and allows the driving fluid (oil) to be pressed into the cartridge. Using this driving mechanism, well-controlled flow rates between 10 and 200 µL/min can be achieved. The glass syringe is filled from a reservoir container, and excess oil is disposed into a waste container (not shown in Figure 7).

The user applies the sample by inserting a syringe with a needle into an opening at the top of the instrument. The needle pierces a septum on the cartridge (cf. Figure 2i), and the sample can be injected. A spring-loaded PCB with flow front sensors based on reflective light barriers is attached to the cartridge. The position of the flow front sensors matches the liquid level check cavities in the cartridge (cf. Figure 2i), enabling effective detection of liquid arrival at these positions. This PCB also contacts the on-board heating resistor integrated in the cartridge. Using a temperature sensor attached to the bottom of the cartridge, the liquids in the intermediate reservoirs can be heated to a defined temperature.

A spring-loaded connector unit with 16 pogo pins establishes electrical contacts to the cartridge PCB for controlling the integrated PIC. The integrated NTC thermistor and a resistor at the back of the PIC allow temperature control of the VCSEL. This prevents signal shifts due to temperature changes during the measurement.

**Figure 8:**
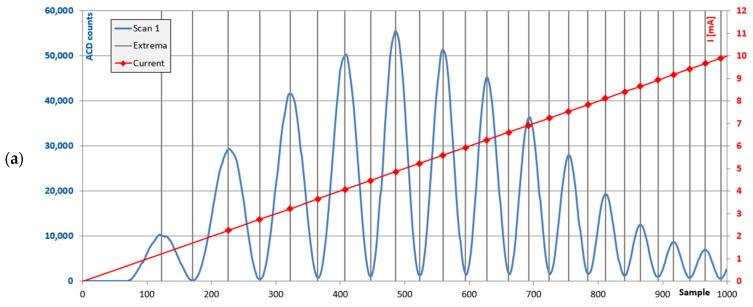

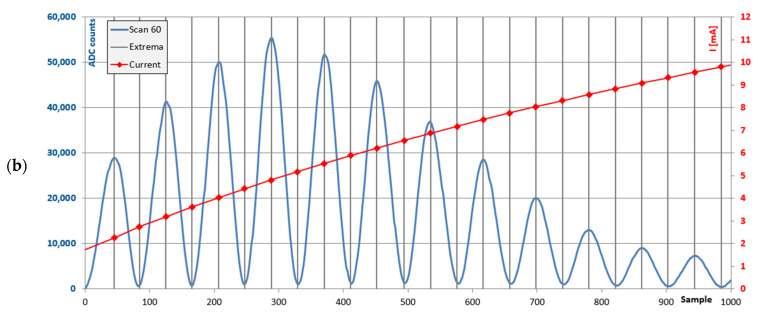
Scans from one photodiode with current profile. (**a**) Before calibration. (**b**) After calibration.

The 850 nm VCSEL is driven with a sawtooth-shaped current profile (0–10 mA) with a frequency of 10 Hz. The emission wavelength of the VCSEL depends on the applied current, and the current increase corresponds to a change of the emission wavelength of approximately 3 nm near its nominal value of 850 nm. However, the current–wavelength dependency is non-linear, resulting in an interference pattern with decreasing distance between the maxima; see Figure 8a. Therefore, the signal of one of the auxiliary channels is analyzed to find the current values for each maximum and minimum. A new profile is created by distributing these current values equidistantly over time; see Figure 8b. This profile is then used to drive the VCSEL when liquids are running over the PIC, thus resulting in linear wavelength scans and corresponding transmission spectra.

The 8 PDs on the PIC are read out with a sample rate of 1000 samples per scan, resulting in the recording of an aMZI transmission spectrum for each PD for each wavelength scan. As explained above, refractive index changes near the PIC surface cause a phase shift of the transmission spectrum. This phase shift is tracked by a Fourier transform-based algorithm. Thus, the phase shift of each aMZI biosensor is recorded as a function of time, and this sensorgram is presented to the user for further data analysis and interpretation.

**Figure 9:**
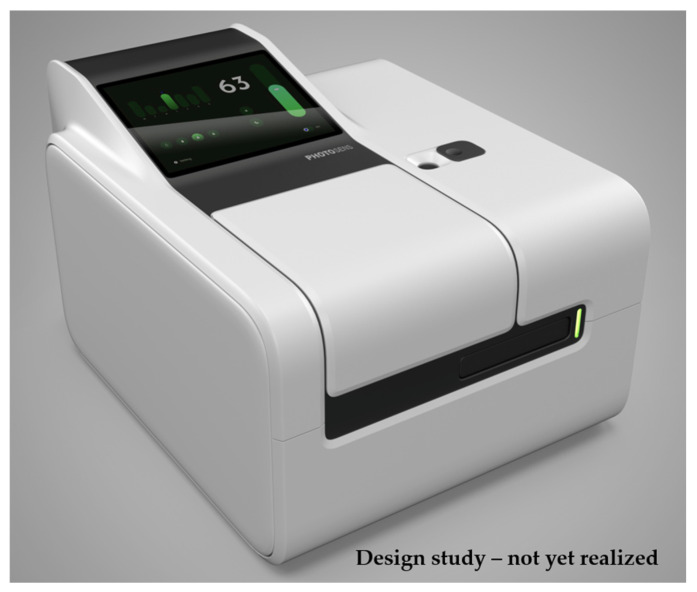
Industrial design study for an integrated instrument that is compatible with the currently developed prototype microfluidic cartridge and readout instrument as shown in Figure 7.

The low-level control of all functions is performed by the instrument’s main PCB (not shown in Figure 7). Basic application software installed on a separate PC is used for high-level configuration and control of cartridge processing. A future stand-alone instrument would have all functions integrated, including advanced software for data analysis and interpretation, and a user-friendly (graphical) interface, e.g., based on a touch screen. Figure 9 presents a design study for a commercial instrument and gives an impression of what such a future product could look like. The instrument design as presented has a footprint of approximately 270 × 380 mm^2^ and a maximum height of 200 mm. The technical design shown in Figure 7a would fit inside this instrument.

### 3.2. Measurement Results

#### 3.2.1. Measurement of Bulk Refractive Index Change Using the Hybrid PIC System

As a first demonstration of the functionality of the full system, the change in bulk refractive index was measured.

**Figure 10:**
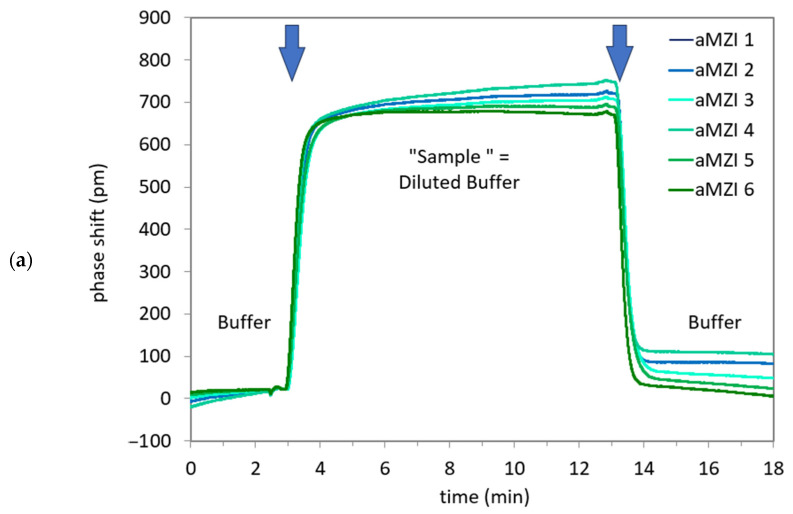

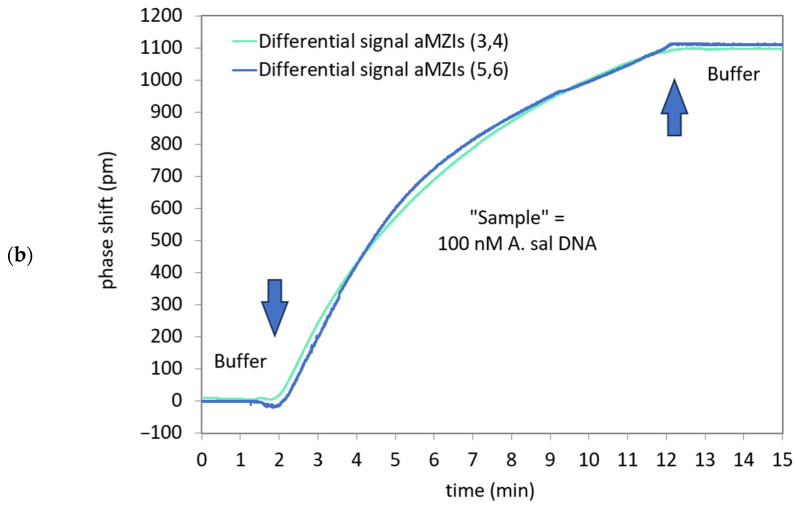
(**a**) Sensorgram for the hybrid PIC during a salt step measurement in which the sensor is exposed to PBS solutions with different NaCl concentrations (Δ[NaCl] = 40 mM). The blue arrows indicate the transitions of the different liquids over the PIC. (**b**) Sensorgram for the hybrid PIC during detection of 100 nM A. sal DNA. Shown are the differential signals between a detection aMZI (aMZIs 4 and 5) and the nearest control aMZI (aMZIs 3 and 6, respectively). The blue arrows indicate the transitions of the different liquids over the PIC.

Temperature control was not used, since this was associated with air bubbles observed on the PIC. When pumping PBS at the start of the measurement, some drift was observed (<10 pm/min), which may be related to the lack of temperature control or swelling and molecule exchange with the chemical coating on the sensors. To facilitate determination of the sensor response to the change in refractive index, the same linear drift correction was applied to all aMZI signals, and the phase shift of all aMZIs was set to zero at the moment the sample reached the PIC. The result of a salt step measurement is shown in Figure 10a.

During exposure to the sample, the signal rises to an average plateau value of 707 ± 25 pm, which is in good agreement with previous measurement results obtained in-house using non-hybrid PICs (data not published). When switching back to PBS, the signal quickly decreases and then levels off. The phenomenon where the signal does not return to the baseline for the aMZI sensors can be attributed to drift and sample dispersion due to diffusion during transport. When a linear drift correction is applied to all aMZI signals, it is important to note that different aMZIs may benefit to varying degrees from this correction. This is because each sensor may exhibit unique characteristics and responses to the correction process. If the drift was not linear prior to the sample injection, applying a linear drift correction may not completely filter out all drift effects. As a result, the signal may not return to the baseline after the sample injection, leading to deviations in the sensor readings. The phase shifts clearly show the expected transitions from buffer to sample and back to buffer, and the measured refractive index sensitivity of approximately 1800 nm/RIU is as expected for this aMZI design, i.e., it is similar to previously obtained results using non-hybrid PICs and in the same range as the theoretical sensitivity for this aMZI design of approximately 1900 nm/RIU. Based on these results, it can be concluded that the system works as expected.

#### 3.2.2. Pathogen DNA Detection Using qPCR

**Figure 11:**
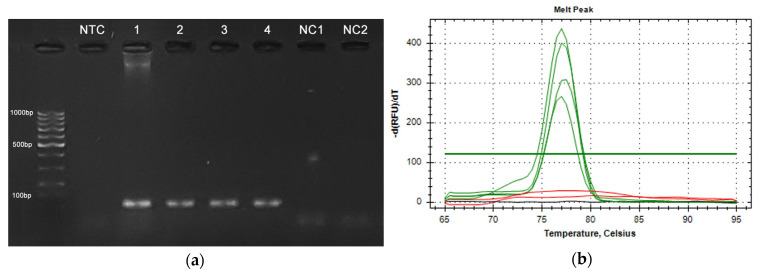
Functionality and specificity confirmation of the A. sal qPCR primer. (**a**) Agarose gel electrophoresis of four A. sal positive samples (1–4), two negative controls (NC1 = salmon DNA, NC2 = V. sal DNA), and a no-template control. (**b**) qPCR melt curve analysis of the identical sample set. Green = A. sal positive samples (1–4), red = negative controls (salmon and V. sal DNA), black = no-template control.

All qPCR-Primer sets were successfully tested for their functionality and specificity by agarose gel electrophoresis and melt curve analysis subsequent to SYBR-qPCR. Exemplary confirmation of primer specificity and functionality for A. sal is shown in Figure 11. In addition, all designed assays (primer–probe sets) were tested and evaluated regarding their specificity and sensitivity. No-template and negative controls were checked for all qPCR assays and were part of each individual qPCR analysis. The quantification threshold was set at a fluorescence intensity of 60 Relative Fluorescence Units (RFUs). Possible non-specific amplification in the presence of non-target DNA, such as salmon DNA and eDNA from different streams and a non-commercial salmon farm, could be excluded by the absence of quantification cycle (Cq) values ≤40 for all three primer–probe sets. The Cq value represents the number of amplification cycles needed to reach the quantification threshold.

**Figure 12:**
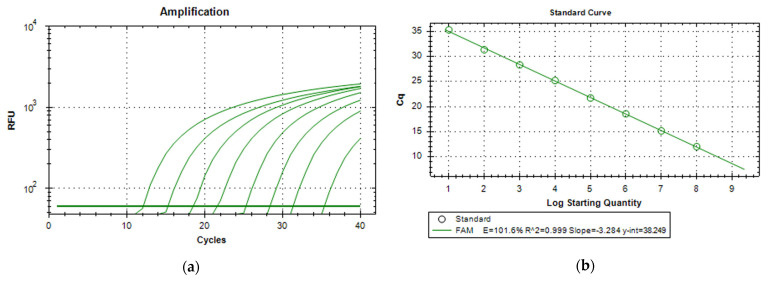
(**a**) Logarithmic amplification plot for a 10-fold dilution series (10^8^ to 10^1^ copies/µL) of the A. sal gBlocks standard. Shown is the mean fluorescence intensity (RFU) as a function of the number of amplification cycles of a duplex measurement. The horizontal line represents the Cq threshold. (**b**) Standard curve of qPCR data relating the Cq value to the starting quantity (copies/µL) of A. sal gBlocks DNA. Samples were analyzed in duplicate, but the standard deviations of the Cq values are too small to be observed in the figure. Efficiency value [E], Slope, y-intercept [y-int], and correlation coefficient [R^2^] values are used to provide information about the performance of the reaction.

Using both synthetic DNA (gBlocks) and extractions of media with known CFU counts of pathogens, a standard series in a range of 10^8^ to 10^1^ copies/µL for the quantification of unknown samples was successfully set up and tested with the respective assay. An averaged duplex amplification diagram and the averaged standard curve for the A. sal *vapA* gene (gBlocks) are shown in Figure 12. The standard curve was linear in the range tested (R^2^ > 0.999) with a slope of −3.284. An amplification efficiency of 101.6% was determined.

Equivalent results were achieved for the Y. ruc and the V. sal assays. Concentrations of down to 10^1^ copies/µL were successfully detected in all assay set-ups. However, in some standard series, no signal (Cq > 40) was measured at concentrations of 1 copy/µL, and therefore the lower detection limit for all assays is estimated to be 5 copies/µL.

Cellulose acetate filtrate samples were found to have a mean A. sal DNA recovery rate of 53.4% (n = 6), while quantifying frozen media equivalents ranging from 10^1^ to 10^6^ CFU. This means that approximately half of the DNA starting quantity could be recovered after Chelex-100 extraction. At the same time, direct DNA extraction of frozen media CFU equivalents with the same extraction method resulted in theoretical recovery rates of 130.3% (n = 10). The quantification results above 100% are mainly due to the presence of dead and inert cells as well as free pathogen DNA in the liquid media. Accordingly, an actual recovery rate of living A. sal cells of approximately 41% can be assumed. The calculated DNA loss appears to be quite high but is still significantly lower than with alternative extraction methods (e.g., only 1.5% recovery rate for V. sal using kit extractions).

Experiments on the storage of cellulose acetate filters previously used for the filtration of CFU equivalents show a clear preference for storage at −20 °C either in the dry state or in ethanol. After 14 days of dry storage at −20 °C, 88.0% of the starting DNA concentration could still be detected, and a recovery rate of 86.0% was found for filters stored in ethanol at the same temperature. In comparison, the recovery rates for filter storage at room temperature after 14 days were only 74.2% (dry) and 78.7% (in ethanol).

#### 3.2.3. Pathogen DNA Detection Using the Hybrid PIC System

With the ability to detect refractive index changes demonstrated, and the results from the qPCR assays, the next challenge was to detect pathogen-specific target sequences of DNA on the hybrid PIC platform.

**Figure 13:**
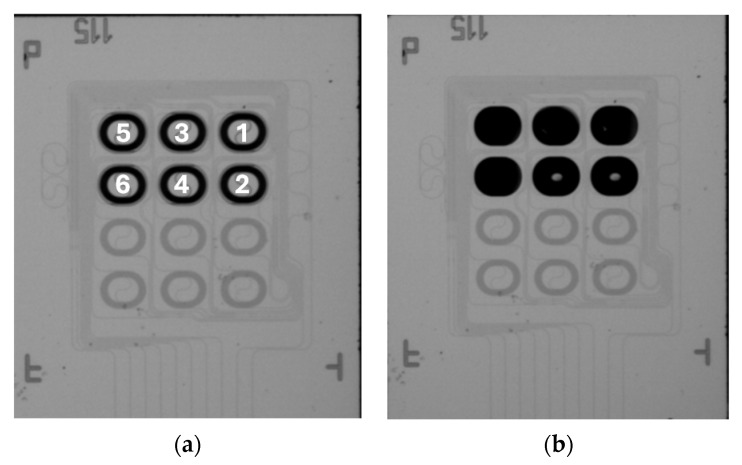
PIC with asymmetric aMZIs used for DNA detection. The numbers indicate the sensing arm of each aMZI. (**a**) Before spotting; (**b**) after spotting of capture and control probes on the sensing arms (‘small’ spirals) of the aMZIs.

Figure 13 shows optical microscopy images of a PIC before and after spotting of the DNA probes (before washing). The lower six spiral waveguides are the reference arms of the unbalanced aMZIs and are covered by silicon oxide, which is why they have a different color in the image. The upper six spiral waveguides are the sensing arms of the aMZIs, three of which are used for detection of A. sal, and three of which are used as controls as described above. After spotting (Figure 13b), the spiral is no longer visible because it is covered by the deposited droplet. It is clearly visible that the spotting solutions have been accurately deposited on the sensing arms, and no mixing between the droplets has occurred.

Hence, aMZIs 1, 4, and 5 were spotted with the A. sal capture probe, while aMZIs 2, 3, and 6 were spotted with the CT47 control probe (numbers refer to Figure 13a). Capture and control probes were spotted on the PIC after it had been integrated in the microfluidic cartridge to minimize the number of processing steps between spotting and measurement. As the sample, a 100 nM solution of single-stranded A. sal DNA (target sequence, complementary to the immobilized capture probe) in a 4x SSC buffer was used. The blisters contained the same buffer, to which ProClin 300 (0.5% *v*/*v*) was added as a preservative to prevent microbial growth during blister storage.

Figure 10b shows the phase shift data of the detection of A. sal DNA. Again, the signal of all channels was set to zero at the beginning of the sample injection. No baseline correction was applied. For this cartridge, an air bubble was observed on top of the aMZIs 1 and 2 at the end of the measurement. This explains why no useful signal could be recorded from these channels. For evaluating the difference between detection and control aMZIs, the signal of a control aMZI was subtracted from the signal of the detection aMZI that is nearest to it on the PIC.

Thus, Figure 10b shows the difference between aMZIs 4 and 3, and between aMZIs 5 and 6. It is observed that the net DNA binding signals of the aMZIs are very similar. A second cartridge yielded similar results. The average net DNA binding signal for 100 nM A. sal is 1107 ± 85 pm, in good agreement with previous in-house measurement results obtained with this sequence using non-hybrid PICs (data not published).

Note that if balanced aMZIs would have been used with the A. sal capture probe immobilized on the sensing arm and the CT47 control probe immobilized on the reference arm, these differential signals would have been obtained directly. By using unbalanced aMZIs, more information is obtained about, e.g., non-specific adsorption as well as changes in bulk refractive index and temperature. On the other hand, two unbalanced aMZIs are needed to measure a net binding signal, which can also be obtained by a single balanced aMZI. Therefore, unbalanced aMZIs are useful during assay development. In this study, unbalanced aMZIs were selected for experimentation due to the limited exploration of sequences (two sequences: A. sal capture probe and CT47 control probe). This choice aimed to provide a deeper understanding of non-specific adsorption on the CT47 control probe. While raw data illustrating this phenomenon are not shown here, it is worth noting that no non-specific adsorption was observed on the CT47 control probe sensors. Balanced aMZIs are, however, preferred once the assay has been optimized, since more biomarkers can be detected simultaneously on a single PIC.

## 4. Conclusions

In summary, in this paper a photonic biosensing platform based on silicon nitride waveguide technology with integrated active components is presented. PIC biosensors are based on interferometric detection of refractive index changes using an aMZI waveguide configuration. Compared to the state of the art [18] and outlined in Table 3, the design and the manufacturing processes of the PIC and the microfluidic cartridge were significantly improved. Moreover, the newly developed process flow includes chemical surface modification and biofunctionalization of PICs with integrated components, which enables the hybrid PIC platform to be used for biosensing for the first time.

Design optimization of the waveguide circuitry and an improved cartridge integration concept enabled a reduction of the PIC footprint by a factor of 8 without sacrificing functionality or sensitivity, resulting (as a first order approximation) in an equivalent reduction of the cost per chip. Also, wafer-scale processes for hybrid integration of components by thermocompression bonding, soldering, and adhesive bonding were developed based on laser-assisted local heating of substrate wafers. Furthermore, novel processes for wafer-scale material-selective chemical surface modification and singulation by stealth dicing were developed. Together, all of these processes constitute a complete and scalable process flow for wafer-scale production of hybrid biosensor PICs.

The hybrid PIC is assembled into a microfluidic cartridge comprising a sample injection port and blister pouches with assay buffers and reagents. The cartridge also features a heating element, degassing functionality, cavities for flow front detection, and a waste reservoir. Moreover, the cartridge provides electrical, mechanical, and fluidic interfaces with the tabletop readout instrument that was developed in parallel. The instrument contains mechanics, electronics, and software for cartridge handling, heating, optical actuation, and readout of the PIC and controlling of liquid flows.

As the first application of the photonic biosensor platform, pathogen detection in aquaculture was chosen. Three important bacterial pathogens for salmon aquaculture (A. sal, V. sal, and Y. ruc) were selected for assay development. DNA biomarkers and corresponding primer–probe sets for these pathogens were designed, and qPCR assays were developed that could detect biomarker concentrations down to 5 copies/µL. The biomarker for A. sal was used for detection using the hybrid PIC platform, demonstrating its potential for biosensing.

Due to the presence of air bubbles on the PIC, some of the sensor spots could not be used. The nucleation point for the air bubbles were the sharply edged sensor cavities. With the use of the on-PIC heating, the formation was increased, and this heater was disabled for the latest experiments. In future, the Surfix Diagnostics (Wageningen, Netherlands) Coat & Close process can be applied, which smooths the edges and thus reduces nucleation points, which has been proven in parallel work (not published).

The hybrid PIC approach meets the urgent demand for disposable, cost-efficient, and user-friendly diagnostic devices with a fast response time and multiplexing potential. PCR and qPCR methods are hampered by being expensive, cumbersome, labor intensive and require complex infrastructure and technical knowhow. All these resources are beyond the capabilities of small fish farmers and are cost-prohibitive for large fish farmers and therefore result in lower numbers for testing, which in turn increases danger to our food security. In conclusion, the results presented in this paper advance the state of the art and contribute to the increasing technology, manufacturing, and market readiness of integrated biophotonics, bringing practical applications of (hybrid) PICs in biosensing one step closer.

## Figures and Tables

**Table 1:** Salmon aquaculture pathogens selected for qPCR assay development.

Pathogen	DSM No.	Target Gene	Primer/Probe 5′-3′ (F: Forward Primer; R: Reverse Primer; P: Probe Sequence)
*Aeromonas salmonicida* (A. sal)	19634	*vapA*	F: TTTCTGGCGTAGGCCGTTTA R: CTTCCGAACGTCATTAACTTCACC P:/6-FAM/CTGCTGGTT/Zen/AACCCGAATGATCATGGTAAT/IABkFQ/
*Vagococcus salmoninarum* (V. sal)	6633	*pheS*	F: AGAGCGGGAAATTCGTCTTC R: GGGAAGGCTGTAACGTTTGTA P:/SUN/ACTGAACCT/Zen/TCCGTCGAAGTTGATGT/IABkFQ
*Yersinia Ruckeri* (Y. ruc)	18506	*glnA*	F: TCCAGCACCAAATACGAAGGT R: GATCTGCGTTCTGCCATGT P:/HEX/CCCAGTTGA/Zen/TTCCGCGCAAGATCT/IABkFQ/

**Table 2:** Target sequence and capture probe used for detection of A. sal on the hybrid PIC.

A. sal Assay	Nucleotide Sequence
Target sequence	3′-GACGACCAATTGGGCTTACTAGTACCATTAT(15)-5′
Capture probe	5′-NH_2_–iSp9-CTGCTGGTTAACCCGAATGATCATGGTAAT-3′

**Table 3:** Comparison of the state-of-the-art cartridge with the presented solution.

	State of the Art	Current Design
Hybrid biosensor PIC	yes	yes
Chip footprint [mm]	10.0 × 12.5	3.0 × 5.0
Chip area [mm^2^]	120	15
Sensing window arrangement	6 × 2 = 12	3 × 4 = 12
Cartridge footprint [mm]	64.0 × 100.0	74.0 × 107.0
Channel cross section towards sensor [mm]	1 × 1	0.5 × 0.44
Including preheating	no	yes
Implementing degassing	no	yes
wafer-level hybrid integration of components into PIC	no	yes
wafer-level anti-fouling coating	no	yes
bio-functionalization compatible with PIC in cartridge integration	no	yes
Scalable process for PIC into cartridge integration	semi	yes
Pre-loading of liquid possible	yes	yes

## Data Availability

The research findings and conclusions are adequately supported by the data presented in the article. Restrictions apply to the availability of data to allow for the commercialization of research findings. Further inquiries can be directed to the corresponding authors.
